# PINIR: a comprehensive information resource for Pin-II type protease inhibitors

**DOI:** 10.1186/s12870-021-03027-0

**Published:** 2021-06-09

**Authors:** Nikhilesh K. Yadav, Nidhi S. Saikhedkar, Ashok P. Giri

**Affiliations:** 1 Publication and Science Communication Unit, CSIR-National Chemical Laboratory, Dr. Homi Bhabha Road, Pune, 411008 India; 2 Information Systems Area, Indian Institute of Management Indore, Indore, 453556 India; 3 Biochemical Sciences Division, CSIR-National Chemical Laboratory, Dr. Homi Bhabha Road, Pune, 411008 India; 4 Academy of Scientific and Innovative Research (AcSIR), Ghaziabad, 201002 India

**Keywords:** Protein sequence analysis, Pin-II type protease inhibitor, Database, Knowledge representation, Annotation, Data analysis

## Abstract

**Background:**

Serine protease inhibitors belonging to the **P**otato type-II **In**hibitor family **P**rotease **I**nhibitors (Pin-II type PIs) are essential plant defense molecules. They are characterized by multiple inhibitory repeat domains, conserved disulfide bond pattern, and a tripeptide reactive center loop. These features of Pin-II type PIs make them potential molecules for protein engineering and designing inhibitors for agricultural and therapeutic applications. However, the diversity in these PIs remains unexplored due to the lack of annotated protein sequences and their functional attributes in the available databases.

**Results:**

We have developed a database, PINIR (Pin-II type PIs Information Resource), by systematic collection and manual annotation of 415 Pin-II type PI protein sequences. For each PI, the number and position for signature sequences are specified: 695 domains, 75 linkers, 63 reactive center loops, and 10 disulfide bond patterns are identified and mapped. Database analysis revealed novel subcategories of PIs, species-correlated occurrence of inhibitory domains, reactive center loops, and disulfide bond patterns. By analyzing linker regions, we predict that alternative processing at linker regions could generate PI variants in the Solanaceae family.

**Conclusion:**

PINIR (https://pinir.ncl.res.in) provides a web interface for browsing and analyzing the protein sequences of Pin-II type PIs. Information about signature sequences, spatio-temporal expression, biochemical properties, gene sequences, and literature references are provided. Analysis of PINIR depicts conserved species-specific features of Pin-II type PI protein sequences. Diversity in the sequence of inhibitory domains and reactive loops directs potential applications to engineer Pin-II type PIs. The PINIR database will serve as a comprehensive information resource for further research into Pin-II type PIs.

**Supplementary Information:**

The online version contains supplementary material available at 10.1186/s12870-021-03027-0.

## Background

Protease inhibitors (PIs) function as intrinsic defense molecules for plants and are gaining importance as therapeutics [1, 2]. **P**otato type **In**hibitor-II family **PI**s (Pin-II type PIs) form a major family of plant PIs, which plays a vital role in protecting plants against biotic stress, particularly against lepidopteran insect pests [3]. Pin-II type PIs are serine PIs found mainly in Solanaceous plants and are induced upon wounding or insect infestation. They inhibit the digestive serine proteases present in the insect gut by forming a protease-PI complex that causes a reduction in protein assimilation and ultimately leads to retardation in the growth and development of the insect [4, 5]. Pin-II type PIs have also been utilized as anti-obesity agents to reduce food intake in humans because these PIs increase the level of satiety hormone (cholecystokinin) via trypsin inhibition [6–8]. The protease inhibitory potential of Pin-II type PIs has also been used for the development of antimicrobial and antifungal agents [9, 10].

**Fig. 1:**
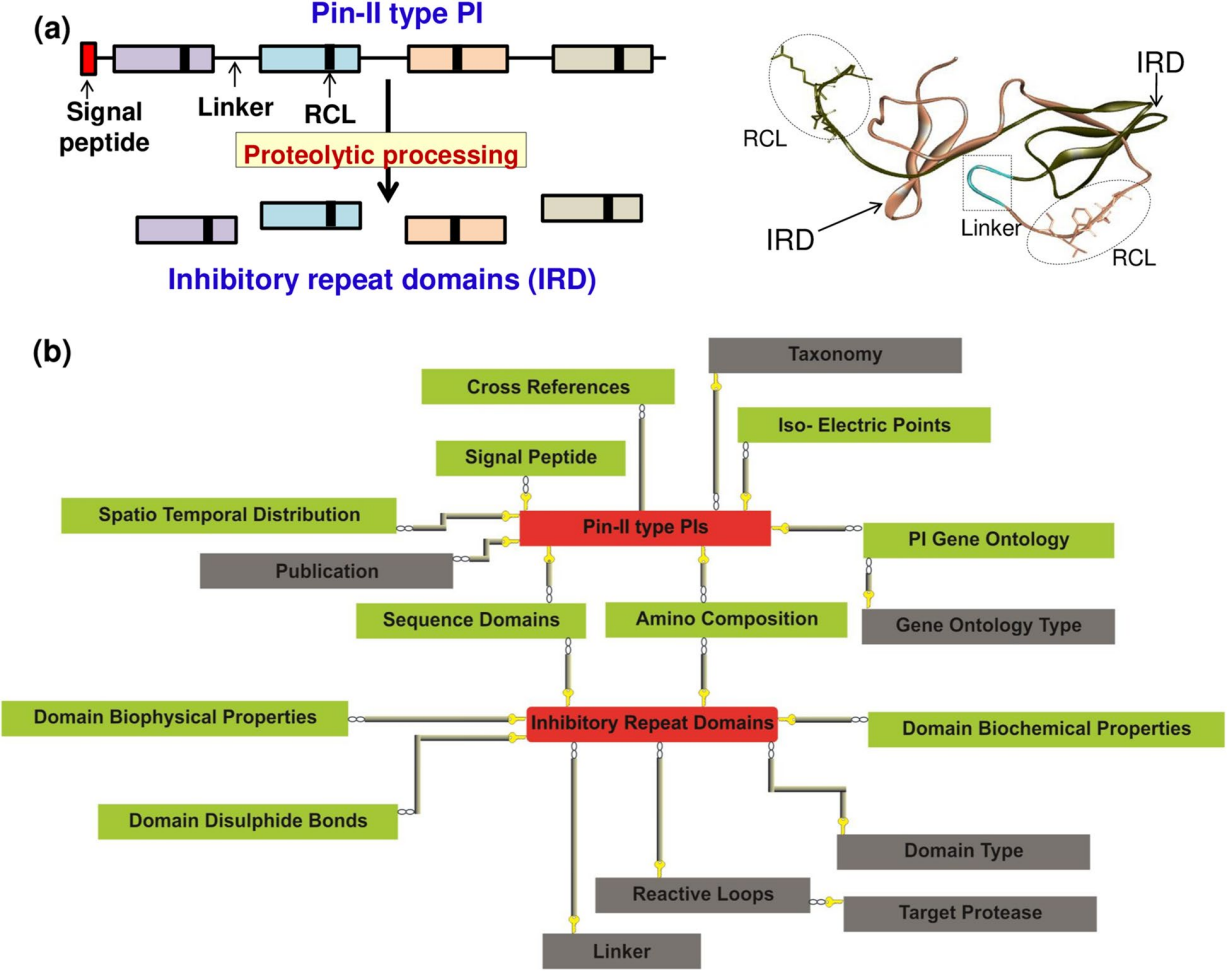
Development of PINIR Database (**a**) Schematic depicting processing of Pin-II type PIs into IRDs (left), and structure of bi-domain Pin-II typePI from tomato (PDB id: 1pju) showing characteristics of Pin-II type PIs- IRD, linker and RCL regions (right). **b** Conceptual Data Model (CDM) of PINIR database, showing the entities and relations between them. All the entities are structured around two Primary entities highlighted within red rectangles (Pin-II type PIs and Inhibitory RepeatDomains). The entities which capture the details about the two primary entities are shown within green rectangles and the grey rectangles represent the entities which provide the support data. The images depicted in the figure are original images prepared by the authors

The unique feature of Pin-II type PIs is the presence of multiple inhibitory repeat domains (IRDs). A single Pin-II type PI consists of multiple IRDs joined by 5 to 6 amino acid linker regions. The IRDs are 50 to 55 amino acid long proteins with a unique structure stabilized by disulfide bonds [11, 12]. Proteolytic processing at linker regions releases IRDs from its parent Pin-II type PI molecule. These IRDs can then target multiple serine proteases [5, 13] (Fig. 1a). The number of IRDs in a PI is variable; also, the protein sequences of IRDs show subtle differences, which may profoundly impact the function of IRDs [14]. For example, IRD-7 and IRD-9 (IRD-145 and IRD-143 in the PINIR database) from *Capsicum annuum* are more than 90% similar in the protein sequence but show a significant difference in the inhibitory activity towards serine proteases [15]. This difference is attributed to the replacement of two cysteine residues in IRD-7 by serine in IRD-9, which causes the loss of two disulfide bonds. These variations result in increased flexibility of IRD-9, allowing a better fit of IRD-9 in the active site of trypsin, and hence higher inhibitory activity [15, 16]. The diversity in protein sequences is useful for combinatorial inhibition since the IRDs could target resistant proteases generated upon adaption to other PI isoforms [4]. Each IRD contains a tripeptide loop called the reactive center loop (RCL), which provides target specificity to the Pin-II type PIs [17]. RCL is the primary interaction site for target serine protease and functions as an inhibitory tripeptide independent of the native IRD scaffold [18].

Pin-II type PIs provide a multi-level strategy to design molecules for agricultural and therapeutic applications. PIs with multiple IRDs joined by short linkers demonstrate a potential approach for simultaneous *in-vitro* delivery of PIs [19, 20]. The disulfide bonded IRD scaffold by itself is a potential candidate for engineering PIs for human therapeutics, as demonstrated for cyclotides [21, 22] and Bowman-Birk protease inhibitor (BBI) proteins [23]. Moreover, since RCL regions define the reactivity of Pin-II type PIs [17], grafting of these peptides on the existing scaffolds can make proteins with altered functions. The RCL tripeptides can also be used as small molecule PIs since they are amenable for chemical peptide synthesis [24].

These signature sequences (IRD, linker and RCL) vary in number and sequence in different Pin-II type PI proteins. Thus, it is imperative to have a dedicated resource of these PIs to understand the classification and diversification of this family. Although several protein and PI databases are available, none provides detailed annotated information for this Pin-II type PI family. Specifically, the number and position of IRDs, Linker, RCL, disulfide linkages and biochemical features are not mapped in any protein databases.

We have developed **PINIR** (Pin-II type PIs Information Resource)**,** available at https://pinir.ncl.res.in, a web-based information system that provides a user-friendly web interface with utilities for browsing and analyzing information related to Pin-II type PIs. The current release of PINIR has sequence annotations of 415 Pin-II PI sequences with a collection of 695 IRDs, 75 Linkers, and 63 RCLs. Biochemical features such as expression pattern, target protease and insects are also compiled in the database. Further, we have categorized the Pin-II type PIs based on the annotations such as position, number and sequences of IRDs, linkers and RCLs. Upon analysis of the PINIR database for linker regions in Pin-II type PI sequences, we hypothesize that the Pin-II type PIs might undergo alternative processing to generate diverse IRDs. Further interpretation of this dataset will highlight the hierarchical categorization of the Pin-II type PI sequences and serve as a useful scientific resource.

## Construction and content

### Database development model

**Fig. 2:**
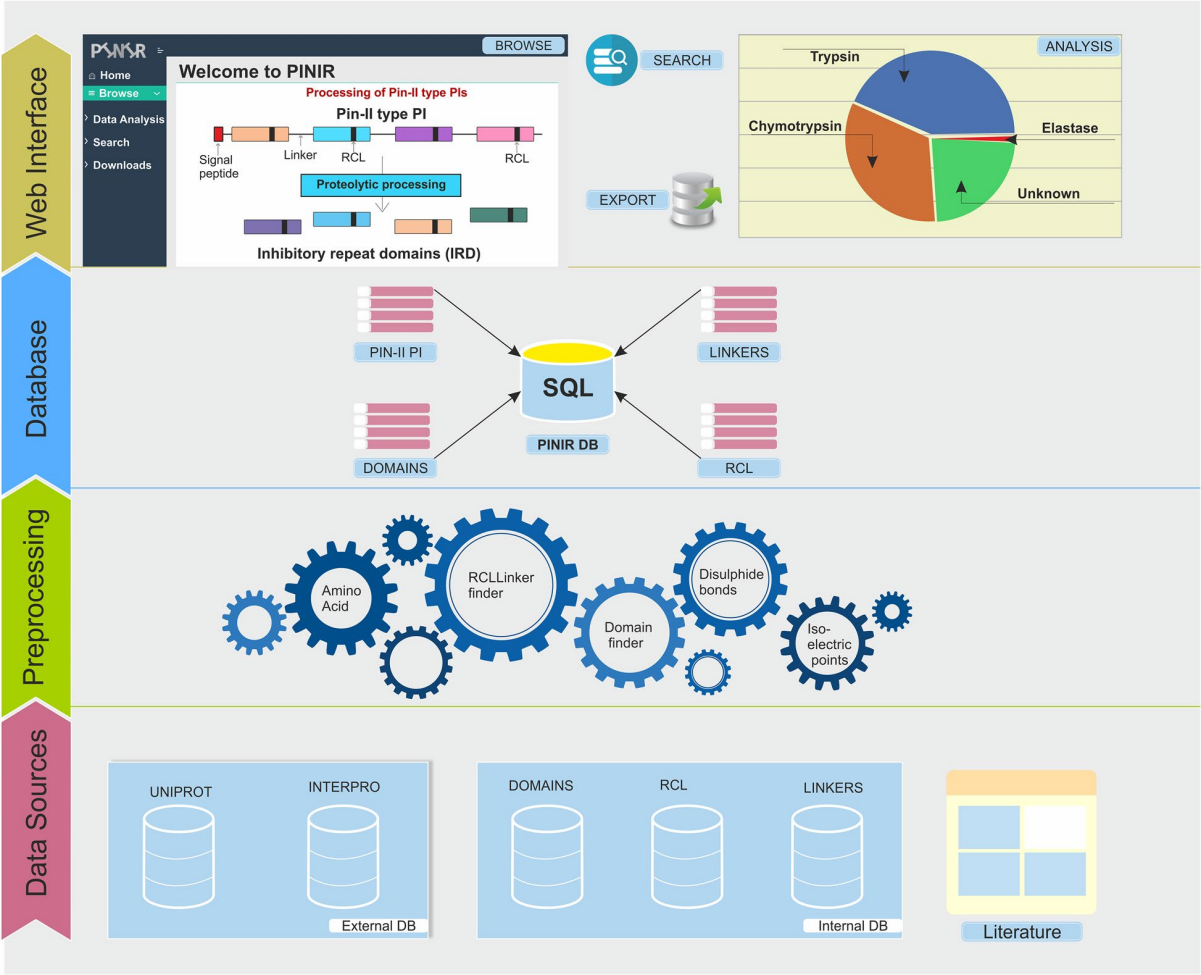
Development Model followed for the creation of PINIR comprises of four development stages: Data Sources, Data Preprocessing and Integration, Database Creation; PINIR Website. The images depicted in the figure are original images prepared by the authors

Literature review of Pin-II type PIs and the study of protein databases resulted in the elucidation of various features of Pin-II type PIs, which determined the various elements of our database (Supplementary Table S1). Figure 1b displays the Conceptual Data Model (CDM) of the PINIR database. It identifies the different entities and relations between them. All the information is structured around two primary entities:'Pin-II type PIs' and 'Inhibitory Repeat Domains' (IRDs/Domains). Table 1 lists the entities which capture the detailed information about the primary entities and those which provide support information. The Development Model followed for the assembling of Pin-II PI information resource is depicted in Fig. 2. It comprises of four development stages: (1) Data Sources; (2) Data Preprocessing and Integration; (3) Database Creation; (4) and PINIR Website.

**Table 1:** Conceptual Data Model (CDM) Entities of PINIR Database

Primary Entities	Entities providing detail information	Entities providing support information
Pin-II PIs	Signal Peptides	Taxonomy
	Iso-electric Points	Publication
	Spatio-Temporal Distribution	Gene Ontology Type
	Cross-References	
	Gene Ontology	
Domains	Sequence Domains	Linkers
	Amino acid Composition	Reactive Loops
	Domain Biophysical Properties	Target Protease
	Domain Biochemical Properties	Domain Type
	Domain Disulfide Bonds

#### Data sources

The data for the entities of the PINIR database scheme was accumulated from several sources such as external databases, published information, and in-house data compilation.

- The protein sequences and associated data about Pin-II type PIs were extracted from Uniprot [25] and Interpro [26]. These online databases were queried for terms belonging to the Pin-II PI family using Interpro ID: IPR003465 and Pfam ID: PF02428. A total of 415 Pin-II PI records were downloaded from Uniprot. Out of 415, 16 were reviewed and 399 were unreviewed Uniprot entries.
- Identification of IRDs in the Pin-II type PIs was performed using HMMSEARCH with Pfam domain ID PF02428, which gave 404 results. PHMMER search was also performed using a few sequences from UniProtKB (P84813, P01078, P01079, P56615, P83241). Removal of redundant and incomplete sequences resulted in a total of 695 IRDs.
- Data for Reactive Center Loops (RCL) and Linkers were manually prepared by sequence alignment of IRD sequences. 75 unique Linker Regions and 63 RCLs were identified in the Pin-II type PI family.
- Publications related to Pin-II type PI were searched using Pubmed IDs from UniProt and Google Scholar. Experimental information such as Spatio-temporal Distribution, Domain Structure Folds, Biochemical Properties, and Biophysical Properties was compiled manually from the articles.

#### Data preprocessing and integration

The preprocessing of the data was either done manually or, in many cases, was automated by using Python programs or Structured Query Language (SQL) queries and procedures. Following are the scenarios where the data preprocessing was carried out:

- The Pin-II type PI data imported from Uniprot had monolithic records that suffered from redundancy and covered varied features of Pin-II type PIs. For efficient processing and creating simplified views, the Uniprot data was distributed into different tables using SQL queries and procedures, keeping their relationships intact.
- The manually prepared list of RCL sequences and their target protease was made more informative by adding P1, P2, and P1` positions of an RCL sequence. Also, the organism field was enriched with Genus and Species data. Both of these were achieved by using programs written in Python.
- Python programs were written to determine the positions of 63 RCL and 75 Linkers in the 695 IRDs (domains).
- Another significant property, "Domain Type" which is determined based upon the position of RCL and Linker in the domain sequence, was identified for all the available IRDs using a Python program.
- A Python program was written to capture the data about the position and occurrence of IRDs in Pin-II PI sequences.
- Information about the disulfide bonds in the IRDs was compiled using the online utility DISULFIND [27]. To identify the Isoelectric Points of Pin-II type PI sequences, the online utility of the website http://isoelectric.org/ [28] was used.
- We used Python programming to derive the information regarding the amino acid composition of Pin-II type PIs and Domain sequences.

#### Database creation

PINIR is a web-based information system to make the Pin-II type PI database easily accessible to the users. This information system is developed using the WISA (Windows, IIS, SQL, and ASP.net) stack. Windows Server 2012 R2 is used as an operating system, SQL Server 2016 for backend database, it is hosted on IIS 8.0 web server, and the front-end is created using ASP.net (C#) Framework 4.7.

**Fig. 3:**
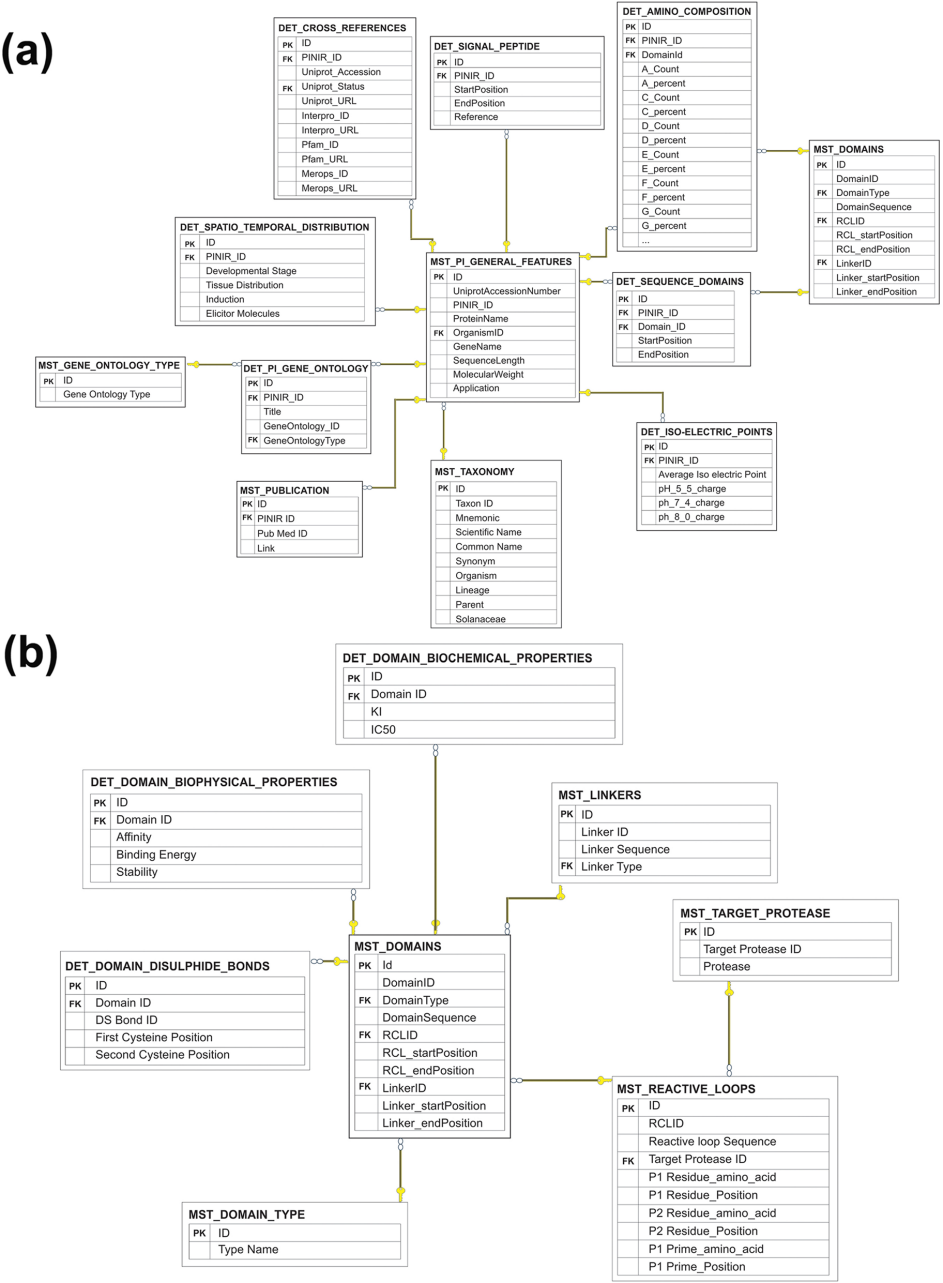
Logical Data Model (LDM) of PINIR. LDM captures entities and relationships and also specifies Attributes, Primary Key and Foreign Keys for each entity. **a** and **b** present the LDM for Primary entities Pin-II type PIs and Inhibitory Repeat Domains, respectively. The images depicted in the figure are original images prepared by the authors

All the entities identified at the CDM level (Fig. 1b) of PINIR DB were converted into corresponding Tables (listed in Table 2) at the Logical Data Model (LDM) level. LDM captures entities and relationships among them and also specifies attributes, primary Key(s) and foreign Key(s) for each entity, Pin-II type PIs (Fig. 3a) and Inhibitory Repeat Domains (Fig. 3b). At the LDM level, the names of the tables containing data that is not dependent on other tables are prefixed with the acronym 'MST' for Master. The tables containing extra information about Master tables are prefixed with the acronym 'DET' for Detail.

**Table 2:** Description of entities and corresponding tables in PINIR

Entity	Table Name	Content
Pin-II PIs	**MST_PI_GENERAL_FEATURES**	Stores general information about properties and composition of Pin-II PIs such as its Uniprot Accession number, Protein Name, Organism, Gene Name etc
Taxonomy	**MST_TAXONOMY**	Stores organism’s details to which Pin- II PIs belongs to. It is captured from Uniprot database
Sequence Domains	**DET_SEQUENCE_DOMAINS**	Captures the PIs and Domains relationship. Stores information about what Domains are found in a PI sequence
Domains	**MST_DOMAINS**	Stores the information about Inhibitory Repeat Domains (IRD) sequences of the Pin–II PI. It includes the IRD sequence, Domain Type and the RCL and Linker sequences found in it
Domain Type	**MST_DOMAIN_TYPE**	Based upon Linker organization in IRD sequences, stores the various Domain Types: Type 1 (H–L Type), Type 2 (L–H Type) and Type 3 (H + L Type)
Reactive Loops	**MST_REACTIVE_LOOPS**	Stores the information about Reactive center loop (Active site loop) appearing in a domain. It includes the Target Protease found and Residues at P1, P2 and P1` position
Target Protease	**MST_TARGET_PROTEASE**	Stores the Probable target protease for the Pin-II inhibitory domain. These are categorized as: Trypsin; Chymotrypsin; Elastase; and Unknown
Linkers	**MST_LINKERS**	Stores the information about various Linker Sequences appearing between two domains in a Pin-II type PI. It includes the Linker sequence and type
Cross References	**DET_CROSS_REFERENCES**	Stores details about other databases which capture information related to PINIR entries
Domain Biochemical Properties	**DET_DOMAIN_BIOCHEMICAL_PROPERTIES**	Captures Inhibitory potential of an IRD
Domain Biophysical Properties	**DET_DOMAIN_BIOPHYSICAL_PROPERTIES**	Captures Biophysical binding parameters for IRD and protease
Domain Disulfide Bonds	**DET_DOMAIN_DISULFIDE_BONDS**	Captures the occurrence and position of disulfide bonds in a domain
Iso-Electric Points	**DET_ISO-ELECTRIC_POINTS**	Captures the Isoelectric points details of the Pin-II type PI sequences, which is the pH at which a particular molecule carries no net electrical charge
Gene Ontology	**DET_PI_GENE_ONTOLOGY**	Stores the lists of selected terms derived from the GO project, which gives the Functional attributes of Pin-II type PI
Gene Ontology Type	**MST_GENE_ONTOLOGY_TYPE**	Stores the three categories of Gene Ontology: Cellular Component; Biological Process; and Molecular Function
Signal Peptide	**DET_SIGNAL_PEPTIDE**	Captures the localization signal for the Pin-II type PI sequences
Spatio Temporal Distribution	**DET_SPATIO_TEMPORAL_DISTRIBUTION**	Captures the localization and tissue specific occurrence of Pin-II type PI
Publication	**MST_PUBLICATION**	This table stores the information related to references to publications related to PINIR entries
Amino Composition	**DET_AMINO_COMPOSITION**	It stores the information about Amino Acids composition of Pin-II type PI and Domain sequences, which includes the percentage and number of an amino acid present in a sequence

In the LDM, the table MST_PI_GENERAL_FEATURES holds information that identifies the entity 'Pin-II type PIs' in CDM. As shown in Table S2, the attribute 'ID' represents the Primary Key, and 'OrganismID' is the Foreign Key to table MST_TAXONOMY. Similarly, the other primary entity, 'Inhibitory Repeat Domains,' is represented by table MST_DOMAINS in the LDM (Table S2), which holds the information that identifies the IRDs of Pin-II type PIs. Here the 'ID' attribute represents the Primary Key, and there are 3 Foreign Keys, 'RCLID', 'LinkerID', and 'DomainType', which refers to MST_REACTIVE_LOOPS, MST_LINKERS and MST_DOMAIN_TYPE tables, respectively.

Finally, the LDM is converted into Physical Data Model (PDM), representing how the Model is built in the database. In the PDM, additional fields, namely column name, column data type, column constraints, Primary Key(s) and Foreign Key(s) are added to the tables identified in LDM for corresponding entities. Table S2 lists the various table structures for the PINIR database, which is implemented using Microsoft SQL Server.

#### PINIR website

**Fig. 4:**
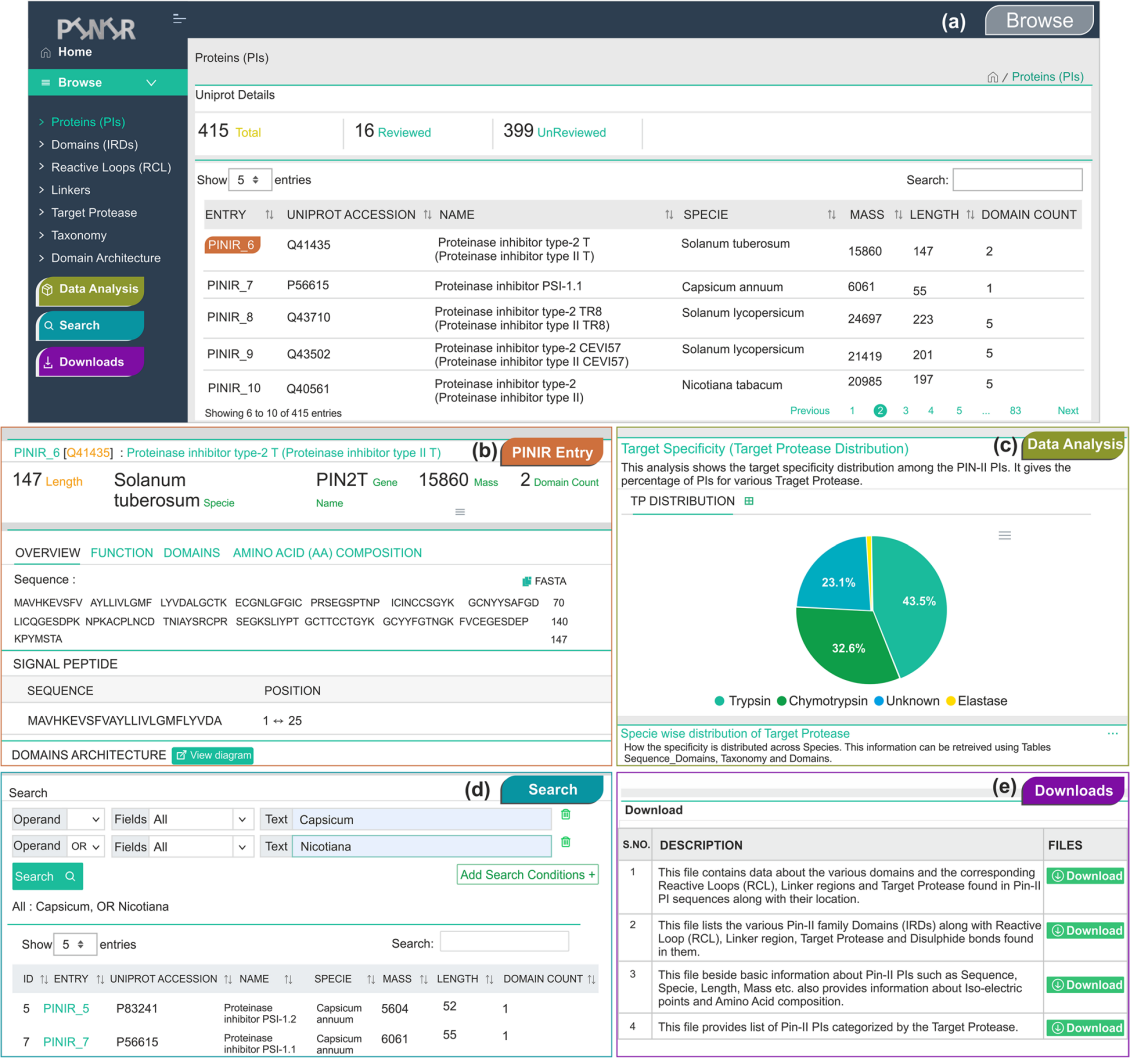
Main features of PINIR website (**a**) Browse page of PINIR (**b**) The Detail page showing the detailed information of a PI (**c**) Data Analysis page provides a comprehensive analysis of the Pin-II type PIs (**d**) Search page of PINIR allows users to search Pin-II type PIs by a variety of keywords (**e**) Download page allows the users to download the PINIR data. The images depicted in the figure are prepared and compiled by the authors using original screenshots of PINIR website

To make the Pin-II type PI database publicly accessible, a web-based information system is developed which can be accessed using: https://pinir.ncl.res.in. PINIR provides an intuitive and user-friendly web interface to Browse, Analyze, Search and Download the information related to Pin-II type PIs (Fig. 4).

#### Browse

The Browse module of the PINIR website provides a user-friendly interface to view the functional annotations of Pin-II type PIssuch as IRDs, RCL, Linker regions, Target Protease(s) and the Organism. Browse by protein, lists a table with PINIR Entry, UniProt Accession Number, Name, Species, Mass, Length and Domains Count. Clicking any Pin-II type PI entry directs to the PIs detail page, composed of four sections: Overview, Function, Domains and Amino Acid Composition.

The Overview section provides basic information about Pin-II type PIs such as Length, Mass, Species, Gene Name, Isoelectric Points and Domains Count. It gives details about a PI sequence, namely, Amino Acid Sequence, Signal Peptide, Domain Architecture. It also provides Cross References to Uniprot, Pfam, InterPro, EMBL, and MEROPS database and links to any References available.

The Function section gives information about the RCL, Target Protease and the GO terms associated with the Pin-II type PI. Details about Spatio-temporal Distribution, Domain Structure Folds, Biochemical Properties, and Biophysical Properties are also provided in this section.

The Domain section lists the IRDs found in the Pin-II type PIs sequence with the RCL and Linkers present in them. It also gives details about the Domain Architecture of the PI sequence.

The Amino Acid composition section lists each amino acid number and its percentage composition in the Pin-II type PIs sequence.

#### Data analysis

The Data Analysis module of the PINIR website aims to provide a comprehensive analysis of the Pin-II type PI database to the users. It is divided into 5 categories: data analysis based on Pin-II type PIs, Domains (IRDs), Reactive Loops (RCL), Linker, and Target Protease. Under each category, the data is analyzed in various ways. For each analysis, a title and a brief description of the results is provided with corresponding charts. These charts are downloadable in PNG (Portable Network Graphics) and SVG (Scalable Vector Graphics) formats, and the result data displayed in tabular format can be downloaded in CSV (Comma Separated Values) file for offline analysis.

#### Search and download

For each of the options in the Browsing module, the results are displayed in a table format. The results data can be sorted by clicking the column header as per the user's requirement. Also, a Quick Search is provided at the top right of the search results table. This Quick Search is case insensitive and accepts any fuzzy term using which users can filter the results based on the content of displayed columns. Apart from this Quick Search, a dedicated Search module is provided on the website. This search allows for text-based search using Uniprot accession, Species, RCL, Linker, Target Protease, and Gene Ontology Terms. Users can also create complex search queries by combining multiple queries using Boolean expressions (e.g., AND, OR and NOT). The search results are displayed in a table, in which each record represents a Pin-II type PI and its information in separate columns. PINIR website also provides a Download module to facilitate its users in downloading the information captured in the PINIR database in CSV files.

## Utility and discussion

Currently available protein and PI databases such as Uniprot, Pfam, Interpro, Plant PI and MEROPS have limited annotated information of protein sequences of the Pin-II type PI family. Hence, we have developed a comprehensive information resource of this family to provide functional annotations of each available Pin-II type PI sequence. We have identified the IRDs, RCL, Linker and Disulfide Bonds and their position in the PI sequences. Physiochemical properties such as isoelectric points and amino acid composition are also compiled within the database. The following sections describe an in-depth data analysis of the PINIR database:

### Occurrence and domain distribution of Pin-II type PI sequences

As of 2nd October 2020, the PINIR database encapsulates information about 415 Pin-II type PIs distributed in 109 species. Out of 415 sequences, 277 Pin-II type sequences are from Solanaceae plants and 138 are from non-Solanaceae plants. A large number of Pin-II type PI sequences are represented by the Solanaceous plant *Capsicum annuum* (*n* = 99), followed by *Solanum tuberosum*(*n* = 52) and *Nicotiana tabacum* (*n* = 22). We also found Pin-II type PI sequences from non-Solanaceae plants, like *Selaginella moellendorffii*, *Coffea canephora* (Rubiaceae), *Rosa chinensis* (Rosaceae), indicating a widespread occurrence of Pin-II type PIs (Supplementary Fig. S1a, b).

We obtained 695 unique IRD sequences in 415 Pin-II type PIs, suggesting that the PIs consist of multiple combinations of IRDs. Despite a higher number of Pin-II type PIs found in genus *Capsicum* than *Nicotiana*, the number of unique IRDs found in the latter was higher, as shown in Supplementary Fig. S1c,d. This shows that the genus *Nicotiana* has a greater diversity of IRD sequences, while *Capsicum* generates a higher number of Pin-II type PIs from a limited set of IRDs. The majority of the IRDs appeared as a part of only one sequence (single occurrence in the database) (Supplementary Table S3). This suggests that the IRDs which are recurrent might be more conserved. Interestingly, all the IRDs were specific to their genus of origin; that is, each domain was found (either once or repeatedly) only in a single genus. Thus, Pin-II type PIs are composed of combinations of IRDs, which might have resulted from the restricted evolution of sequences within a particular genus (Supplementary Table S4).

We found 14 domain architectures of Pin-II type PIs in the PINIR database (Supplementary Table S5). We categorized the Pin-II type PIs as single-domain (n-domains = 1) or multi-domain (n-domains > 1). Among the multi-domain PIs, the majority consisted of n-domains between 2 to 5, and a few n-domains greater than 7. There are 17 domain architectures for Pin-II type PIs in InterPro, of which 9 are composed of domain IPR003465 (Pin-II type PIs), whereas 8 architectures consist of Pin-II domains in combination with domains from other families. Therefore, we identified novel domain architectures for Pin-II type PIs from the PINIR database. Also, Solanaceae plants consisted of PIs with n-domains ranging from 1 to 21, whereas non-Solanaceae plants consisted only of single-domain PIs. Out of 170 single-domain PIs, 60 are distributed among 10 Solanaceae species, whereas 110 single-domain PIs are spread across 63 non-Solanaceae species. This indicates that the Solanaceous plants preferably express multi-domain PIs, which could accommodate different/same IRD sequences needed to inhibit multiple proteases. We also observed that *Capsicum annuum* has a majority of sequences with n-domain = 5, *Solanum tuberosum* has maximum sequences with *n* = 2 or 3, and *Nicotiana tabacum* has maximum sequences with n-domain > 5 suggesting a correlation between n-domains and species (Supplementary Table S6).

### Linker diversity and distribution in Pin-II type PIs

**Fig. 5:**
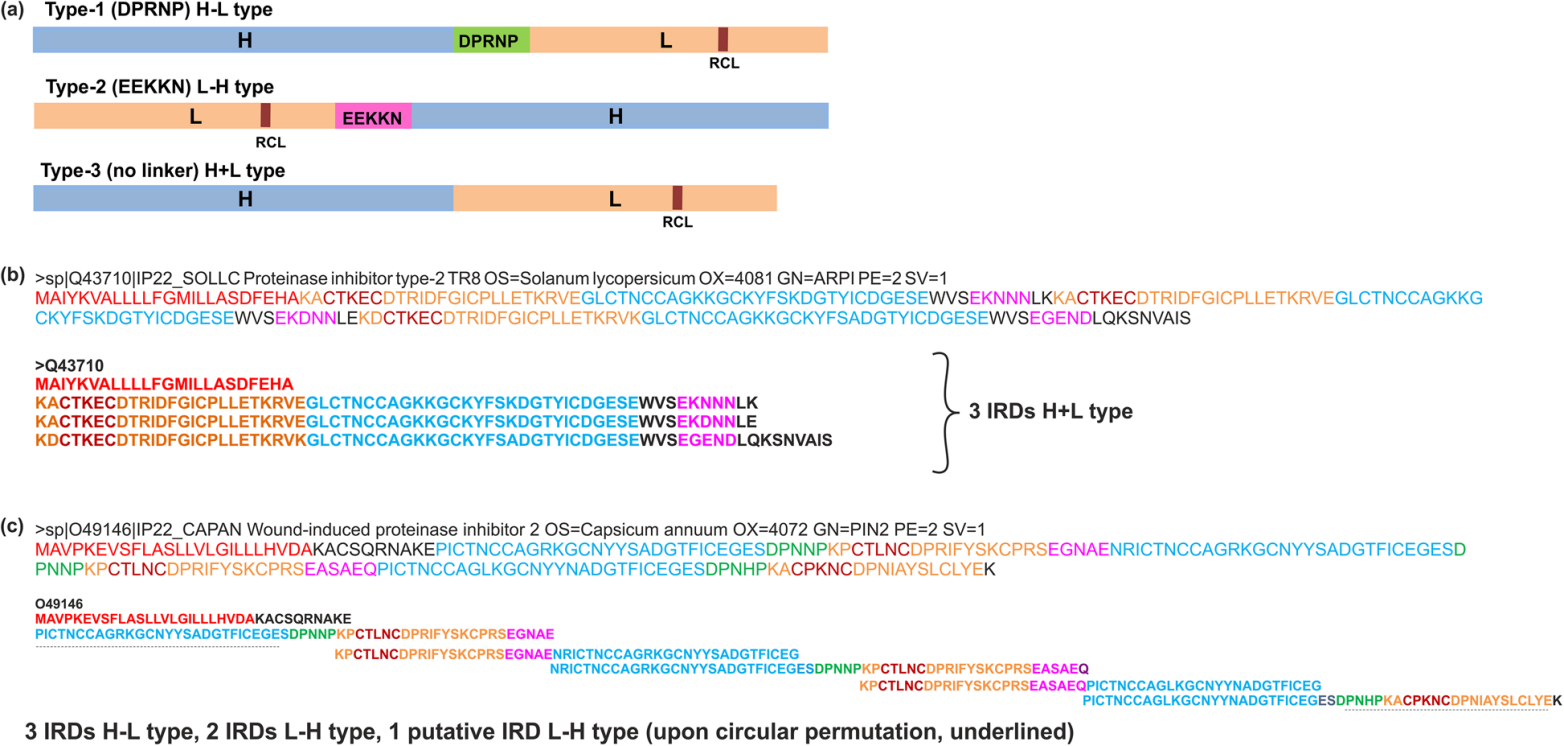
Linker regions in Pin-II type PIs. **a** Classification of IRD sequences based on linker regions; heavy chain (H), light chain (L), RCL and linker regions are color-coded for sequences in (**b**) and (**c**). **b** Example of Pin-II type PI with H + L type IRDs. **c** Example of Pin-II type PI with overlapping H–L and L–H type IRDs. The images depicted in the figure are original images prepared by the authors

Two types of linker regions are present in the Pin-II type PI family, namely type-I (DPRNP) and type-II (EEKKN) [29]. The linker region divides the IRD into heavy (H) and the light fragment (L). The L-fragment consists of the RCL (Fig. 5a). There are three types of IRDs depending on the arrangement of H and L fragments around the linker regions, as shown in Fig. 5a. IRDs with type-I linkers have H–L type arrangement: {H-linker-L}, while those with type-II linkers have L–H type:{L-linker-H}. IRDs without a linker have H + L arrangement (type-III). Upon manual identification of linker regions, we found 75 linker sequences, which belonged to type-I (n = 24) and type-II (*n* = 51).

We classified the IRD sequences into three subtypes, types based on whether type-I, type-II, or no linker region is present in the IRDs. We identified 238 type-I linkers containing IRDs (H–L type), 256 type-II linkers containing IRDs (L–H type) and 201 IRDs with no linker regions (H + L type). Kong and Ranganathan [29] suggested that the presence of the IRD subtype is based upon the domain organization in the multi-domain Pin-II type PIs. Two types of domain organizations are reported for the Pin-II type PI family: tandem repeats (beads on string) or circularly permuted (clasped bracelet). The tandem repeat PIs have L–H type IRDs, whereas, in the circularly permuted PIs, the domain formed by N- and C-terminal sequence is present in the H + L topology, and other domains adopt H–L topology. In our analysis, however, we found that both the L–H and H–L topologies are present within the same IRD sequence, depending on the position of the linker in the parent Pin-II type PI sequence. We mapped the IRD sequences and linker regions to the Pin-II type PI sequences and found that the same fragment can be a part of both (L–H and H–L) topologies upon alternate processing at linker regions. For the H + L type, the linker regions were present outside the IRD sequence (Fig. 5b). The presence of overlapping IRD sequences in the Pin-II type PIs indicates that alternate proteolytic processing of PIs at the linker regions might generate enormous diversity of IRDs from the same parent Pin-II type PI. For example, in Fig. 5c, alternative processing at the linker regions can result in five IRD variants instead of 4 IRDs. The presence of overlapping variant sequences in Pin-II type PIs can be viewed as a natural permutation mechanism to generate vast diversity of IRDs without increasing the protein length. This feature of Pin-II type PIs might be beneficial in co-evolution with insect proteases where multiple proteases could be targeted by several IRDs in the insect gut [30]. It might also explain the higher number of IRDs compared to PIs, as seen in our analysis. Whether these alternate processing mechanisms occur in nature and play a role in Pin-II type PI organization needs to be elucidated.

Species distribution of linker regions showed that IRDs from Solanaceous plants had linker regions, whereas PIs from non-solanaceous plants were all type-III that did not contain linkers. Furthermore, type-I linker containing IRDs are present mainly in *Capsicum*, whereas IRDs with type-II and type-III linkers are found in *Nicotiana* and *Solanum* genera, indicating a preferential occurrence of the linkers depending on the species (Supplementary Table S7).

Furthermore, the Type-I linker sequence DPNNP was exclusive to *Capsicum* species, while DPRNP was found majorly in *Nicotiana* and DPKNP in *Solanum* plants. DPRNP and DPKNP showed widespread occurrence across species. Among the type-II linker sequences, EGNAE was exclusive to *Capsicum*, EEKKN was in *Nicotiana,* and EGSPE in *Solanum* species (Supplementary Fig. S2; Supplementary Table S8, S9). This shows the restricted distribution of linkers, which shows possible species-specific proteolytic processing machinery for Pin-II type PIs.

### RCL distribution and specificity

**Fig. 6:**
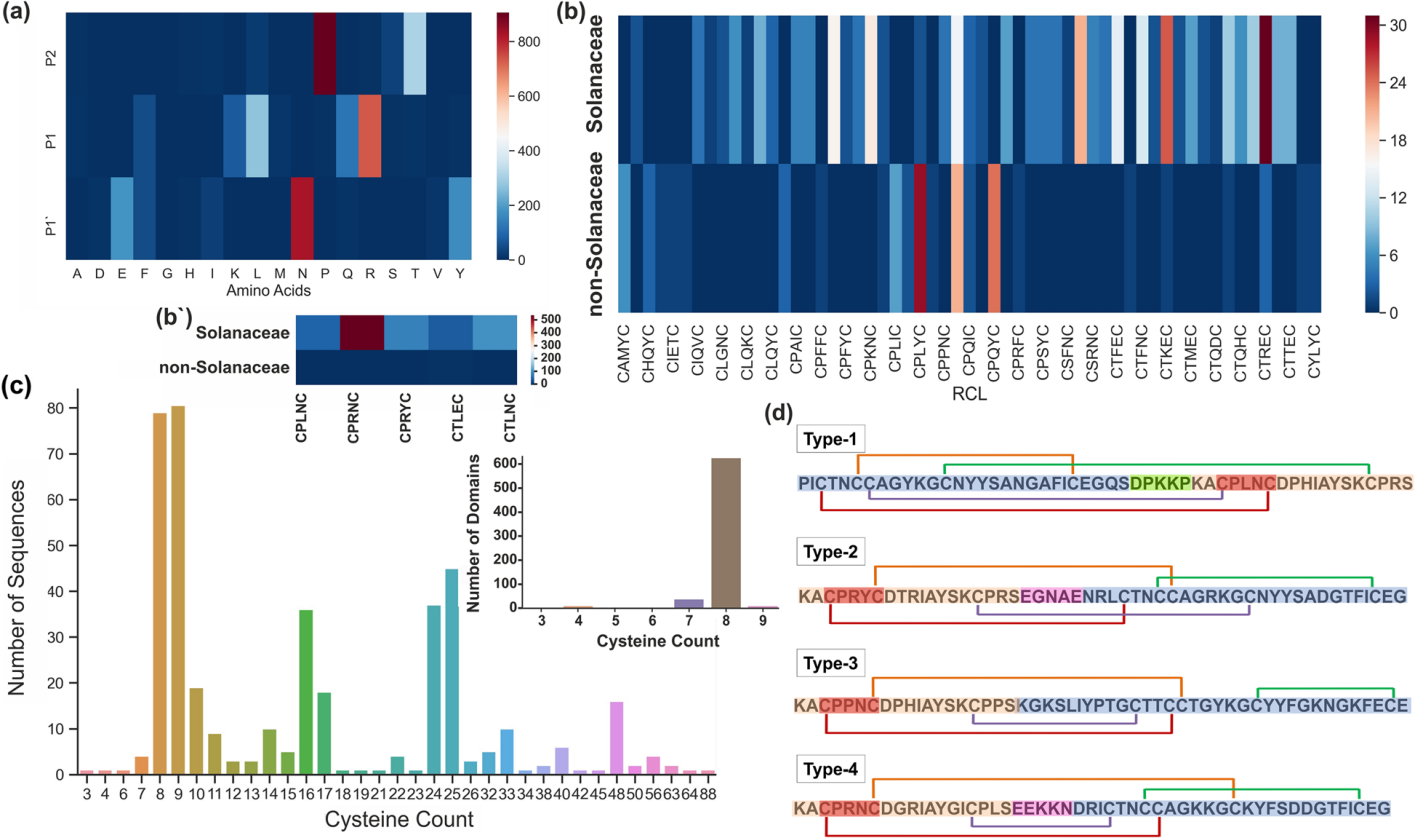
RCL regions and disulfide variants in Pin-II type PIs. **a** Preference of amino acids at P1, P2 and P1’ residues in the RCL regions (**b**) Distribution of RCL sequences across solanaceous and non-solanaceous plants. Frequently occurring RCL are shown separately in (**b**’) (**c**) Cysteine content (number of cysteine residues per sequence) in Pin-II type PIs and IRDs (inset) (**d**) Major types of disulfide bond architectures in IRDs. The images depicted in the figure are original images prepared by the authors

In the IRD sequence dataset, 63 RCL sequences were identified. The RCL sequence "CPRNC" is the most predominant in Pin-II type PIs, occurring 576 times in the Pin-II type PI sequences, followed by "CTLNC" (113 times) and "CPRYC" (94 times) (Supplementary Table S10) while in the IRD dataset, "CPRNC" occurred 291 times. “CPRNC” shows a widespread occurrence in *Capsicum*, *Nicotiana* and *Solanum* species and is found in high numbers. However, “CTLNC” and “CPRYC” display limited occurrence, predominantly in *Capsicum annuum* (Supplementary Table S10). Furthermore, P1', P1 and P2 positions of the RCL interact with the active site of target protease. The distribution of amino acids at these positions shows a preference for a few amino acids at these crucial positions. Specifically, Pro is dominant at P2 residue, followed by Thr (Fig. 6a). It is reported that P2 Pro plays an essential role in determining the potency and specificity of the RCL [18]. Interestingly, Ser is rarely found at this position despite being structurally similar to Thr. At the P1 position, which is the specificity-determining residue, Arg is dominant, indicating that the majority are trypsin inhibitors. This is followed by Leu, indicating chymotrypsin inhibitors. Surprisingly, we found that Gln also occupied P1 position in ~ 100 sequences. The presence of Gln at the P1 site of serine protease inhibitors has not been reported earlier. 23.1% of the targets to the IRDs were unknown due to amino acids like Gln, Ser, Thr, Met, Glu, and Ala at the P1 residue. This might be a unique feature of Pin-II type PIs, leading to diversification of its target specificities. Species distribution of RCLs showed that Solanaceous plants contained diverse RCL sequences, with Arg/Lys/Leu/Gln at the P1 residue. In contrast, non-solanaceous plants majorly contain Gln/Leu at the P1 position (Fig. 6b and 6b'). At P1' residue, Asn was the most common, followed by Tyr and Glu. Elucidation of functional features of these amino acids requires further research.

### Disulfide bond variations in the Pin-II type PI family

Disulfide bonds are crucial for maintaining the proper structural fold for Pin-II type PIs and important for the potency of IRDs [11, 16]. The variations in disulfide bonds and the number of cysteine residues impart differential potency to the Pin-II type PIs [14]. The presence of 4 disulfide bonds is characteristic of IRDs of the Pin-II type PIs. We performed amino acid composition analysis of Pin-II type PI sequences and show that Cys is the most abundant residue, followed by Gly (Supplementary Fig. S3). Other amino acids show a dispersed distribution across the Pin-II type PI family. Analysis of the number of cysteines across the IRDs indicated that most IRDs contain 8 Cys residues or 4 disulfide bonds. Few sequences (*n* = 4) had 7 Cys residues, indicating disulfide bond variants (Fig. 6c).

Further, Cys distribution across the Pin-II type PI sequences showed that most sequences had 8 or 9 Cys, corresponding to that observed for IRDs. Also, sequences with 16, 24, 32, 40 and 48 Cys were prominent, showing multi-domain PIs. Interestingly, sequences with 9,10, 17, 25 and 33 Cys are also present in high numbers, indicating the presence of disulfide bond variants or free Cys residues in the Pin-II sequences (Fig. 6c). Although IRDs consist of 8 Cys residues, the presence of an odd number of Cys in Pin-II type PIs indicates additional Cys-mediated structural scaffolds.

Further, we analyzed disulfide bonds within the IRD sequences. We found 10 types of disulfide bond architectures in the IRDs (Supplementary Table S11). Types 1 to 4 are the most common (Fig. 6d). The disulfide bond types correlated with the type of linker regions, wherein type 1 and type 2 disulfide bond architectures were found predominantly in IRDs with type-1 and 2 linkers, respectively (Supplementary Table S12). Thus, disulfide bond connections are conserved to the type of IRDs. Furthermore, *Capsicum* species comprised type-1 bond architecture, while type 2 and 3 were commonly found in *Nicotiana* species, highlighting a species-specific preference of disulfide bond scaffolds (Supplementary Table S13). In addition to intra-IRD bonds, it will be interesting to determine the inter-IRD disulfide bond connections in Pin-II type PIs.

## Conclusion

Pin-II type PIs have promising applications as scaffolds for protein engineering. These proteins are useful as agricultural pest control agents because they effectively reduce the growth of agricultural insect pests like *Helicoverpa armigera* [4, 15]. Also, the application of RCL peptides as synthesized in linear and cyclic forms has been reported for developing eco-friendly insect pest control strategies [24]. Pin-II PIs have also been used as antimicrobial and antifungal agents [9]. For example, Potamin-I inhibits several proteases like chymotrypsin, trypsin, and papain and is active against pathogenic microbial strains, including *Candida albicans*, *Rhizoctonia solani* [10]. These PIs are also used as formulations for reducing obesity [6, 31]. While only Pin-II type PIs from potatoes have been studied, Pin-II type PIs from other species can also be explored by utilizing the PINIR database, which provides a repository of signature sequences, namely, RCL, linker and domain regions. These annotations are not available in any other public databases, making PINIR a unique resource for research into the Pin-II type plant PI family.

**Fig. 7:**
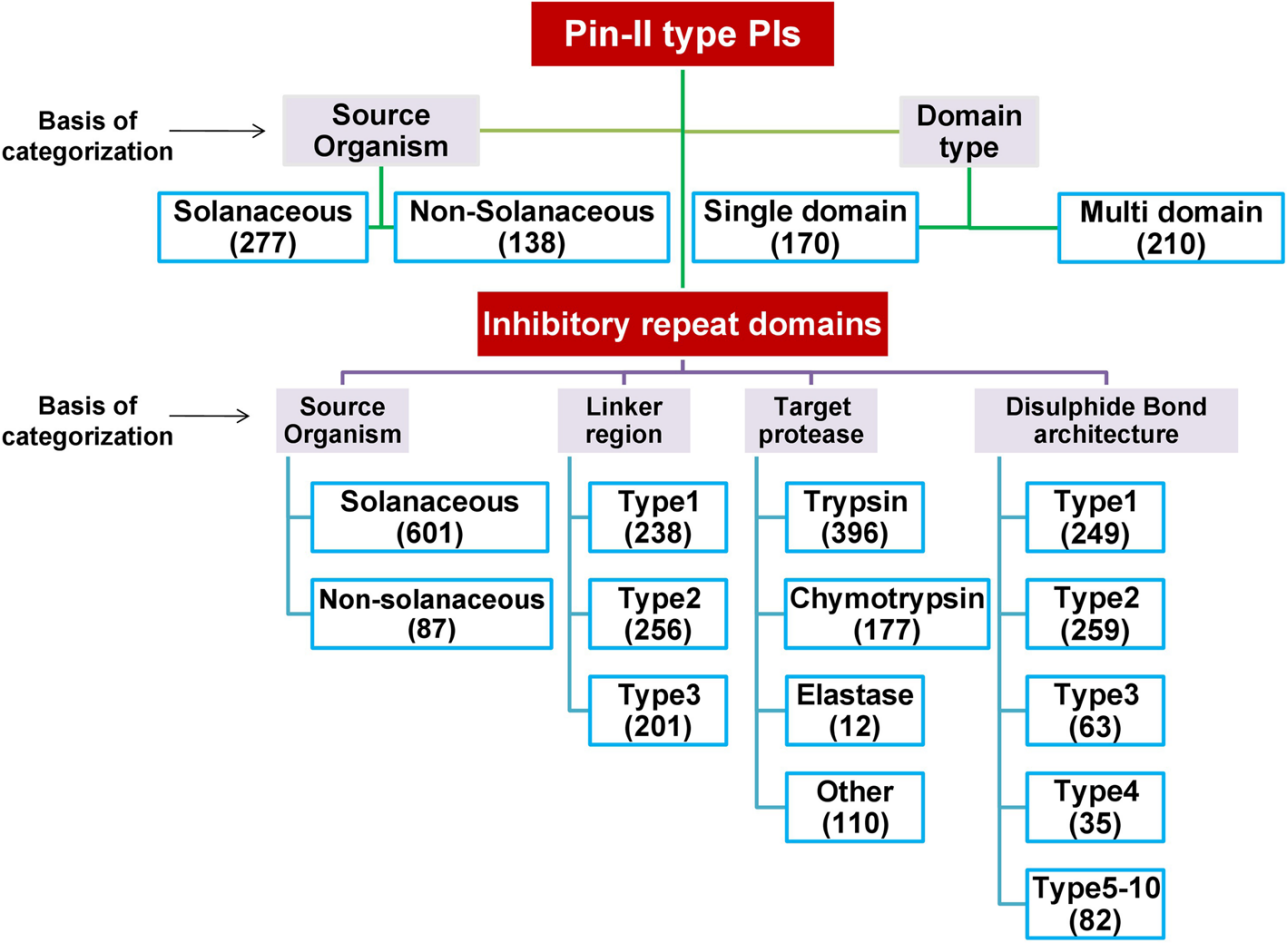
Categorization of Pin-II type PIs in PINIR according to sequence characteristics. Purple boxes represent the basis of categorization, blue boxes represent the categories. The number of sequences in a particular category is mentioned in brackets. The images depicted in the figure are original images prepared by the authors

PINIR is a family-specific database, and it identifies novel subcategories within the Pin-II type PI family based on sequence characteristics (Fig. 7). We identified 688 IRDs within the 415 Pin-II type PIs, suggesting that many PIs have overlapping IRDs and that these IRDs have small variations in their amino acid sequences. The study of linker regions and their positions highlighted both H–L and L–H type IRDs in a continuous Pin-II type PI sequence. This result suggests an unexplored mechanism in plants for producing several combinations of IRDs from the limited size of Pin-II type PI proteins. We aim to continuously update and expand the PINIR database by routinely adding new features and sequences of Pin-II-type PIs.

## Supplementary Information

**Additional file 1: Figure S1.** Distribution of Pin-II type PIs. **Figure S2.** Occurrence of Type-I and Type-II linker regions. **Figure S3.** Percentage distribution of amino acids in Pin-II PIs. **Table S1.** Available information about the Pin-II type PI sequences in online databases. **Table S2.** Detail Table structures implemented in PINIR database. **Table S3.** Occurrence of IRDs in PINIR database. **Table S4.** Species-wise distribution of IRDs (occurrence frequency >10). **Table S5.** Domain architectures in Pin-II type PI family. **Table S6.** Species distribution of multidomain Pin-II PIs. **Table S7.** Genus-wise distribution of linker regions. **Table S8.** Species distribution of type-I linker regions in Pin-II PIs. **Table S9.** Species distribution of type-II linker regions in Pin-II PIs. **Table S10.** Occurrence and distribution of RCL. **Table S11.** Disulphide bonds architecture in IRDs. **Table S12.** Distribution of IRDs according to dsBond type and correlation with linker types. **Table S13.** Genus-wise distribution of IRDs in dsBond types.

## Data Availability

The datasets generated and analyzed during the current study are available in the PINIR repository, https://pinir.ncl.res.in

## Abbreviations

PIs: Protease Inhibitors
Pin-II type PIs: Potato type-II Inhibitor family Protease Inhibitors
PINIR: Pin-II type PIs Information Resource
IRDs: Inhibitory Repeat Domains
RCL: Reactive Center Loop
BBI: Bowman-Birk Protease Inhibitor
CDM: Conceptual Data Model
SQL: Structured Query Language
LDM: Logical Data Model
PDM: Physical Data Model
PNG: Portable Network Graphics
SVG: Scalable Vector Graphics
CSV: Comma Separated Values
