# Supplementary material for: PINIR: a comprehensive information resource for Pin-II type protease inhibitors
Source: BMC Plant Biol. 2021 Jun 9;21:267. doi: 10.1186/s12870-021-03027-0 (PMC8188708; doi:10.1186/s12870-021-03027-0)
Supplement: Supplementary file 1 — Additional file 1: Figure S1. Distribution of Pin-II type PIs. Figure S2. Occurrence of Type-I and Type-II linker regions. Figure S3. Percentage distribution of amino acids in Pin-II PIs. Table S1. Available information about the Pin-II type PI sequences in online databases. Table S2. Detail Table structures implemented in PINIR database. Table S3. Occurrence of IRDs in PINIR database. Table S4. Species-wise distribution of IRDs (occurrence frequency >10). Table S5. Domain architectures in Pin-II type PI family. Table S6. Species distribution of multidomain Pin-II PIs. Table S7. Genus-wise distribution of linker regions. Table S8. Species distribution of type-I linker regions in Pin-II PIs. Table S9. Species distribution of type-II linker regions in Pin-II PIs. Table S10. Occurrence and distribution of RCL. Table S11. Disulphide bonds architecture in IRDs. Table S12. Distribution of IRDs according to dsBond type and correlation with linker types. Table S13. Genus-wise distribution of IRDs in dsBond types. [file 12870_2021_3027_MOESM1_ESM.pdf]

PINIR: A comprehensive information resource for Pin-II type protease inhibitors

Nikhilesh K. Yadav[a, b#], Nidhi S. Saikhedkar[c,d#], Ashok P. Giri[c,d*]

[a]Publication and Science Communication Unit, CSIR-National Chemical Laboratory, Dr. Homi Bhabha Road, Pune-411008, India

[b]Information Systems Area, Indian Institute of Management Indore, Indore 453556, India

[c]Biochemical Sciences Division, CSIR-National Chemical Laboratory, Dr. Homi Bhabha Road, Pune-411008, India

[d]Academy of Scientific and Innovative Research (AcSIR), Ghaziabad 201002, India

[#]Authors contributed equally

*Corresponding author: Ashok P. Giri, E-mail: ap.giri@ncl.res.in

Supplementary Information

Figure S1: Distribution of Pin-II type PIs. Bar graph represents number of (a) Pin-II type PI sequences and (b) number of IRDs unique to a particular genus in non-solanaceous plants. (c) Bar graph representing number of Pin-II type PI sequences and (d) number of IRDs unique to a particular genus in solanaceous plants.

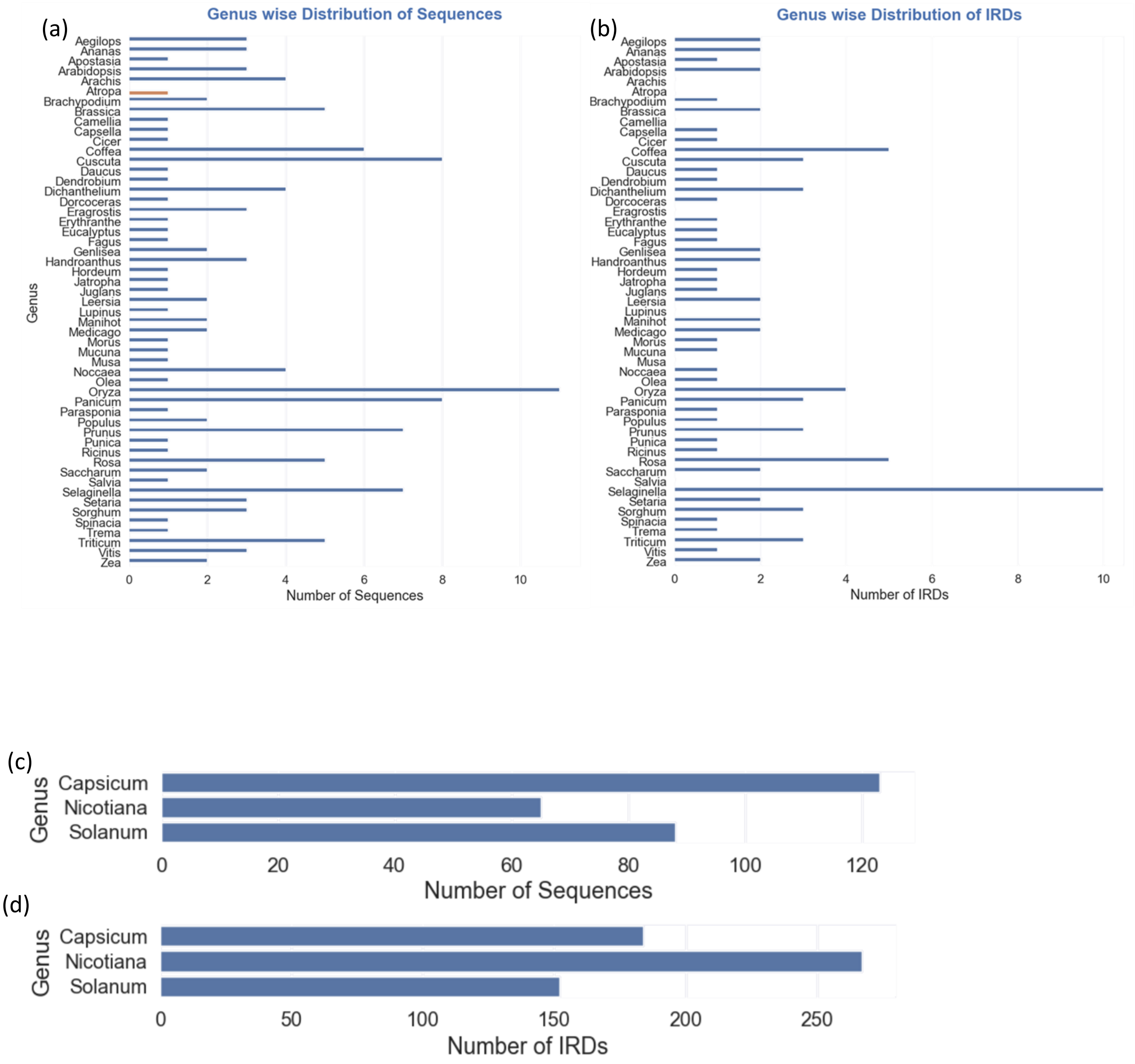

Figure S2: Occurrence of Type-I and Type-II linker regions; Five highly occurring sequences are shown with names of the genus in which they are predominantly found

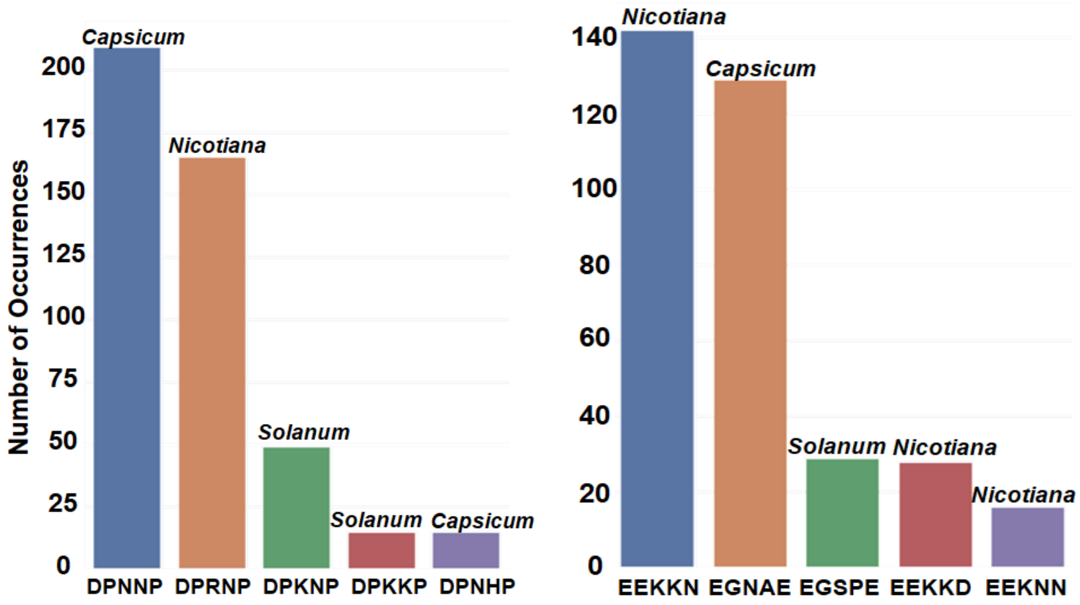

Figure S3: Percentage distribution of amino acids in Pin-II PIs

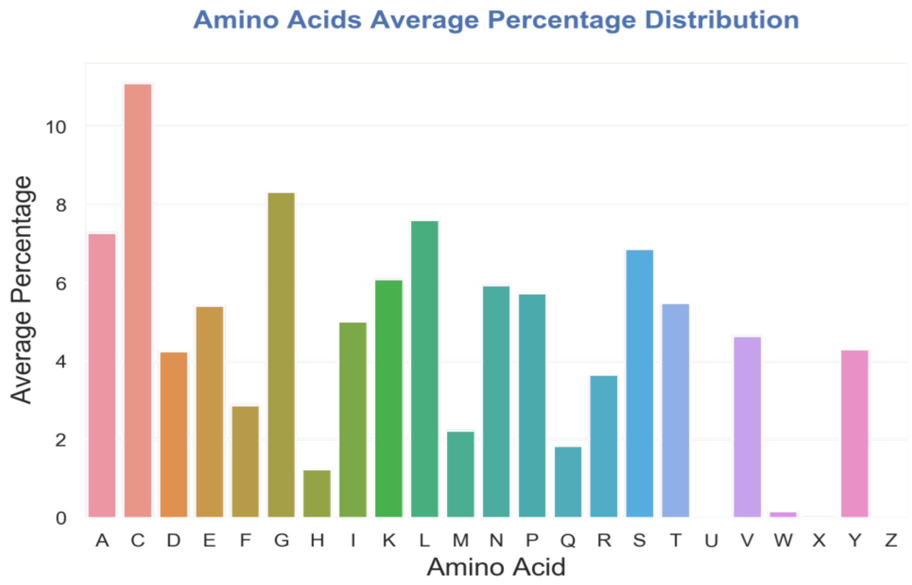

Table S1: Available information about the Pin-II type PI sequences in online databases

Database	Query	Result	Comments
MEROPS [18, 19]	Family identifier: I20	130 sequences with 10 identifiers	Missing sequences
Pfam[20].	Pfam ID: PF02428	309 sequences; 11 domain architectures	Missing sequences; not annotated
InterPro[21]	InterPro ID: IPR003465	417 sequences; 17 domain architectures; 11 structures.	Sequences not annotated for RCL, linkers and disulphide bonds
Plant-PIs[22]	I20 family	55 records	Missing sequences
UniprotKB[23]	MEROPS ID “I20”	41 sequences	Missing sequences
	Pfam ID “PF02426”	415 sequences	Sequences not annotated
	InterPro ID “IPR003465”	415 sequences	Sequences not annotated

Table S2: Detail Table structures implemented in PINIR database

MST_PI_GENERAL_FEATURES

Attribute Name	Data Type	Allow Null	Key	Primary Table
Id	int (4)		Primary Key	
UniprotAccessionNumber	varchar (20)			
PINIR_ID	varchar (20)			
ProteinName	varchar (200)	YES		
OrganismID	int (4)		Foreign Key	Taxonomy
GeneName	varchar (200)	YES		
Sequence	varchar MAX)	YES		
SequenceLength	int (4)	YES		
MolecularWeight	int (4)	YES		
Application	varchar (1000)	YES		

MST_TAXONOMY

Attribute Name	Data Type	Allow Null	Key	Primary Table
Id	int (4)		Primary Key	
TaxonID	int (4)			
Mnemonic	varchar (45)	YES		
ScientificName	varchar (200)	YES		
CommonName	varchar (100)	YES		
Synonym	varchar (200)	YES		
Organism	varchar (200)			
Lineage	varchar (1000)	YES		
Parent	int (4)	YES		
Solanaceae	bit (1)	YES		

DET_SEQUENCE_DOMAINS

Attribute Name	Data Type	Allow Null	Key	Primary Table
Id	int (4)		Primary Key	
PINIR_ID	int (4)		Foreign Key	PI_GeneralFeatures
Domain_ID	int (4)		Foreign Key	Domains
StartPosition	int (4)	YES		
EndPosition	int (4)	YES		

MST_DOMAINS

Attribute Name	Data Type	Allow Null	Key	Primary Table
Id	int (4)		Primary Key	
DomainID	varchar (50)			

DomainType	int (4)		Foreign Key	DomainType
DomainSequence	varchar (1000)	YES		
RCLID	int (4)		Foreign Key	ReactiveLoops
RCL_startPosition	int (4)	YES		
RCL_endPosition	int (4)	YES		
LinkerID	int (4)		Foreign Key	Linkers
Linker_startPosition	int (4)	YES		
Linker_endPosition	int (4)	YES		

MST_DOMAIN_TYPE

Attribute Name	Data Type	Allow Null	Key	Primary Table
Id	int (4)		Primary Key	
TypeName	varchar (255)			

MST_REACTIVE_LOOPS

Attribute Name	Data Type	Allow Null	Key	Primary Table
Id	int (4)		Primary Key	
RCLID	varchar (10)			
ReactiveloopSequence	varchar (10)	YES		
TargetProteaseID	int (4)		Foreign Key	TargetProtease
P1Residue_amino_acid	char (1)	YES		
P1Residue_Position	int (4)	YES		
P2Residue_amino_acid	char (1)	YES		
P2Residue_Position	int (4)	YES		
P1Prime_amino_acid	char (1)	YES		
P1Prime_Position	int (4)	YES		

MST_TARGET_PROTEASE

Attribute Name	Data Type	Allow Null	Key	Primary Table
Id	int (4)		Primary Key	
TargetProteaseID	varchar (10)			
Protease	varchar (50)	YES		

MST_LINKERS

Attribute Name	Data Type	Allow Null	Key	Primary Table
Id	int (4)		Primary Key	
LinkerID	varchar (10)			
LinkerSequence	varchar (10)	YES		
LinkerType	int (4)		Foreign Key	LinkerType

DET_CROSS_REFERENCES

Attribute Name	Data Type	Allow Null	Key	Primary Table
Id	int (4)		Primary Key	
PINIR_ID	int (4)		Foreign Key	GeneralFeatures
Uniprot_Accession	varchar (20)	YES		
Uniprot_Status	varchar (20)	YES	Foreign Key	UniprotStatus
Uniprot_Url	varchar (100)	YES		
Interpro_Id	varchar (20)	YES		
Interpro_Url	varchar (100)	YES		
Pfam_Id	varchar (20)	YES		
Pfam_Url	varchar (100)	YES		
Merops_Id	varchar (20)	YES		
Merops_URL	varchar(100)	YES		

DET_DOMAIN_BIOCHEMICAL_PROPERTIES

Attribute Name	Data Type	Allow Null	Key	Primary Table
Id	int (4)		Primary Key	
DomainID	int (4)		Foreign Key	Domains
KI	varchar (250)	YES		
IC50	varchar (250)	YES		

DET_DOMAIN_BIOPHYSICAL_PROPERTIES

Attribute Name	Data Type	Allow Null	Key	Primary Table
Id	int (4)		Primary Key	
DomainID	int (4)		Foreign Key	Domains
Affinity	varchar (250)	YES		
BindingEnergy	float (8)	YES		
Stability	varchar (250)	YES		

DET_DOMAIN_DISULPHIDE_BONDS

Attribute Name	Data Type	Allow Null	Key	Primary Table
Id	int (4)		Primary Key	
DomainID	int (4)		Foreign Key	Domains
DSBondID	int (4)			
FirstCysteinePosition	int (4)	YES		
SecondCysteinePosition	int (4)	YES		

DET_ISO-ELECTRIC_POINTS

Attribute Name	Data Type	Allow Null	Key	Primary Table
Id	int (4)		Primary Key	
PINIR_ID	int (4)		Foreign Key	PI_GeneralFeatures
AverageIsoelectricPoint	float (8)	YES		
pH_5_5_charge	float (8)	YES		
ph_7_4_charge	float (8)	YES		
ph_8_0_charge	float (8)	YES		

DET_PI_GENE_ONTOLOGY

Attribute Name	Data Type	Allow Null	Key	Primary Table
Id	int (4)		Primary Key	
PINIR_ID	int (4)		Foreign Key	PI_GeneralFeatures
Title	varchar (250)	YES		
GeneOntology_ID	varchar (20)			
GeneOntologyType	varchar (50)		Foreign Key	GeneOntologyType

MST_GENE_ONTOLOGY_TYPE

Attribute Name	Data Type	Allow Null	Key	Primary Table
Id	int (4)		Primary Key	
GeneOntologyType	varchar (255)			

DET_SIGNAL_PEPTIDE

Attribute Name	Data Type	Allow Null	Key	Primary Table
Id	int (4)		Primary Key	
PINIR_ID	int (4)		Foreign Key	PI_GeneralFeatures
StartPosition	int (4)	YES		
EndPosition	int (4)	YES		
Reference	varchar (500)	YES		

DET_SPATIO_TEMPORAL_DISTRIBUTION

Attribute Name	Data Type	Allow Null	Key	Primary Table
Id	int (4)		Primary Key	
PINIR_ID	int (4)		Foreign Key	PI_GeneralFeatures
DevelopmentalStage	varchar (200)	YES		
TissueDistribution	varchar (200)	YES		
Induction	varchar (200)	YES		
ElicitorMolecules	varchar (200)	YES		

MST_PUBLICATION

Attribute Name	Data Type	Allow Null	Key	Primary Table
Id	int (4)		Primary Key	
PINIR_ID	int (4)		Foreign Key	PI_GeneralFeatures
PubMedID	int (4)	YES		
Link	varchar (100)	YES		

DET_AMINO_COMPOSITION

Attribute Name	Data Type	Allow Null	Key	Primary Table
Id	int (4)		Primary Key	
PINIR_ID	int (4)		Foreign Key	PI_GeneralFeatures
DomainId	int (4)		Foreign Key	Domains
A_Count	int (4)	YES		
A_percent	float (8)	YES		
C_Count	int (4)	YES		
C_percent	float (8)	YES		
D_Count	int (4)	YES		
D_percent	float (8)	YES		
E_Count	int (4)	YES		
E_percent	float (8)	YES		
F_Count	int (4)	YES		
F_percent	float (8)	YES		
G_Count	int (4)	YES		
G_percent	float (8)	YES		
H_Count	int (4)	YES		
H_percent	float (8)	YES		
I_Count	int (4)	YES		
I_percent	float (8)	YES		
K_Count	int (4)	YES		
K_percent	float (8)	YES		
L_Count	int (4)	YES		
L_percent	float (8)	YES		
M_Count	int (4)	YES		
M_percent	float (8)	YES		
N_Count	int (4)	YES		
N_percent	float (8)	YES		
O_Count	int (4)	YES		
O_percent	float (8)	YES		
P_Count	int (4)	YES		

P_percent	float (8)	YES		
Q_Count	int (4)	YES		
Q_percent	float (8)	YES		
R_Count	int (4)	YES		
R_percent	float (8)	YES		
S_Count	int (4)	YES		
S_percent	float (8)	YES		
T_Count	int (4)	YES		
T_percent	float (8)	YES		
U_Cont	int (4)	YES		
U_percent	float (8)	YES		
V_Count	int (4)	YES		
V_percent	float (8)	YES		
W_Count	int (4)	YES		
W_percent	float (8)	YES		
X_Count	int (4)	YES		
X_percent	float (8)	YES		
Y_Count	int (4)	YES		
Y_percent	float (8)	YES		
Z_Count	int (4)	YES		
Z_percent	float (8)	YES		

Table S3: Occurrence of IRDs in PINIR database

No. Of Occurrences	No. of IRDs	% of total
1	494	71.8
2-10	180	26.0
11-20	10	1.44
>20	4	0.57

Table S4: Species-wise distribution of IRDs (occurrence frequency >10)

Domains	Species	Number of Occurrences
IRD-133	*Capsicum annuum, Capsicum baccatum*	39
IRD-84	*Capsicum annuum*	29
IRD-536	*Capsicum annuum*	25
IRD-559	*Capsicum annuum, Capsicum baccatum*	23
IRD-122	*Capsicum annuum, Capsicum chinense*	19
IRD-93	*Capsicum annuum, Capsicum baccatum*	18
IRD-560	*Capsicum annuum*	17
IRD-551	*Capsicum annuum, Capsicum chinense*	15
IRD-83	*Capsicum annuum, Capsicum baccatum*	15
IRD-152	*Nicotiana sylvestris, Nicotianatabacum*	14
IRD-121	*Capsicum annuum*	13
IRD-541	*Capsicum annuum*	12
IRD-90	*Capsicum annuum*	12
IRD-143	*Capsicum annuum, Capsicum chinense*	11
IRD-467	*Solanum tuberosum, Solanum chacoense, Solanum phureja*	11

Table S5: Domain architectures in Pin-II type PI family

n-Domains	No. of Pin-II type PIs in PINIR	No. of Pin-II type PIs in InterPro
1	170	199
2	22	79
3	50	81
4	10	16
5	77	7
6	1	19
7	16	4
8	0	3
9	6	0
10	2	0
11	17	1
12	1	0
13	4	0
15	3	0
21	1	0

Table S6: Species distribution of multidomain Pin-II PIs

Organism	n-Domains	Number of Pin-II Pis
Capsicum annuum	5	55
	1	16
	3	16
	7	9
	4	1
	11	1
Nicotiana tabacum	1	8
	11	6
	5	2
	3	1
	6	1
	7	1
	10	1
	12	1
Solanum tuberosum	2	16
	3	15
	1	12
	5	5
	4	4

Table S7: Genus-wise distribution of linker regions

Genus	Type-I IRD	Type-II IRD	Type-III IRD
Capsicum	84	65	34
Nicotiana	114	136	17
Solanum	39	53	59

Table S8: Species distribution of type-I linker regions in Pin-II PIs

Linker sequence	Species	No. of Occurrences
DPNNP	*Capsicum annuum*	196
	Capsicum baccatum	6
	Capsicum chinense	6
DPRNP	*Nicotiana tabacum*	38
	Nicotiana sylvestris	23
	Nicotiana attenuate	18
	Nicotiana obtusifolia	10
	Nicotiana acuminate	6
	Nicotiana rustica	6
	Nicotiana alata	5
	Nicotiana clevelandii	5
	Nicotiana occidentalis	5
	Nicotiana pauciflora	5
	Nicotiana quadrivalvis	5
	Nicotiana glutinosa	4
	Nicotiana megalosiphon	4
	Nicotiana miersii	4
	Nicotiana repanda	4
	Nicotiana simulans	4
	Nicotiana umbratica	4
	Nicotiana benthamiana	3
	Nicotiana linearis	3
	Nicotiana spegazzinii	2
	Solanum tuberosum	2
	Solanum americanum	1
	Solanum cavicola	1
	Solanum chacoense	1
	Nicotiana corymbosa	1
	Solanum lycopersicum	1
	Solanum Solanumnigrum	1
DPKNP	*Solanum tuberosum*	17
	Capsicum annuum	6
	Nicotiana glutinosa	4
	Nicotiana alata	3
	Capsicum chinense	3
	Solanum lycopersicum	2
	Nicotiana obtusifolia	2
	Nicotiana annuum	2
	Solanum americanum	1
	Nicotiana attenuate	1
	Capsicum baccatum	1
	Solanum chacoense	1
	Nicotiana linearis	1
	Nicotiana miersii	1
	Solanum nigrum	1
	Solanum phureja	1
	Nicotiana spegazzinii	1
	Nicotiana sylvestris	1
DPKKP	*Solanum tuberosum*	13
	Solanum chacoense	1
	Solanum lycopersicum	1
DPNHP	*Capsicum annuum*	13
	Capsicum baccatum	1
	Capsicum chinense	1

Table S9: Species distribution of type-II linker regions in Pin-II PIs

Linker sequence	Species name	No. of occurrences
EEKKN	*Nicotiana tabacum*	25
	Nicotiana sylvestris	19
	Nicotiana attenuate	18
	Nicotiana alata	10
	Nicotiana obtusifolia	10
	Nicotiana glutinosa	6
	Nicotiana pauciflora	6
	Nicotiana miersii	5
	Nicotiana occidentalis	5
	Nicotiana rustica	5
	Nicotiana umbratica	5
	Nicotiana simulans	4
	Nicotiana benthamiana	3
	Nicotiana clevelandii	3
	Nicotiana megalosiphon	3
	Nicotiana quadrivalvis	3
	Nicotiana repanda	3
	Nicotiana spegazzinii	3
	Nicotiana cavicola	2
	Nicotiana linearis	2
	Nicotiana acuminate	1
	Nicotiana corymbosa	1
EEKKD	*Nicotiana tabacum*	16
	Nicotiana obtusifolia	6
	Nicotiana clevelandii	2
	Nicotiana quadrivalvis	2
	Nicotiana rustica	2
EGNAE	*Capsicum annuum*	123
	Capsicum baccatum	3
	Capsicum chinense	3
EGSPE	*Solanum tuberosum*	20
	Solanum americanum	2
	Solanum chacoense	2
	Solanum lycopersicum	2
	Solanum nigrum	2
	Solanum phureja	1
EEKNN	*Nicotiana glutinosa*	6
	Nicotiana acuminata	2
	Nicotiana attenuata	2
	Nicotiana sylvestris	2
	Nicotiana tabacum	2
	Nicotiana linearis	1
	Nicotiana spegazzinii	1

Table S10: Occurrence and distribution of RCL

RCL	Plant	Occurrence in Pin-II PI	Occurrence in IRDs
**CPRNC**	*Capsicum annuum; Nicotiana tabacum; Nicotiana attenuate; Nicotiana sylvestris; Nicotiana obtusifolia*; *Solanum tuberosum*; *Nicotiana glutinosa*; *Nicotiana acuminate; Nicotiana alata*; *Nicotiana clevelandii*; *Nicotiana quadrivalvis*; *Capsicum chinense*; *Nicotiana miersii*; *Nicotiana pauciflora*; *Nicotiana linearis*; *Nicotia narustica; Nicotiana spegazzinii*; *Solanum lycopersicum*; *Nicotiana megalosiphon*; *Nicotiana occidentalis; Nicotiana simulans*; *Nicotiana umbratica*; *Solanum nigrum*; *Capsicum baccatum; Nicotiana benthamiana*; *Nicotiana corymbosa*; *Nicotiana repanda*; *Solanum phureja*; *Solanum melongena*	576	291
**CPRYC**	*Capsicum annuum*; *Capsicum baccatum; Capsicum chinense*	94	18
**CTLNC**	*Capsicum annuum; Nicotianata bacum*; *Nicotiana alata; Nicotiana sylvestris; Capsicum chinense; Nicotiana rustica*	113	36

Table S11: Disulphide bonds architecture in IRDs

Architecture Type	Bond Categories	Number of IRDs
Type-1	['bond(3,40)', 'bond(6,24)', 'bond(7,36)', 'bond(13,49)']	249
Type-2	['bond(3,28)', 'bond(7,32)', 'bond(16,38)', 'bond(31,49)']	260
Type-3	['bond(3,34)', 'bond(7,35)', 'bond(16,31)', 'bond(41,52)']	63
Type-4	['bond(3,32)', 'bond(7,38)', 'bond(16,28)', 'bond(31,49)']	35
Type-5	['bond(3,27)', 'bond(7,15)', 'bond(29,48)', 'bond(30,37)']	20
Type-6	['bond(3,30)', 'bond(7,15)', 'bond(27,37)', 'bond(29,49)']	8
Type-7	['bond(3,30)', 'bond(7,36)', 'bond(15,48)', 'bond(27,29)']	12
Type-8	With 3 dsBonds	28
Type-9	With 2 dsBonds	15
Type-10	With 1 dsBond	1

Table S12: Distribution of IRDs according to dsBond type and correlation with linker types

dsBond type	No. of IRDs		
	**Linker-Type-1**	**Linker-Type-2**	**Linker-Type-3**
Type-1	216	0	33
Type-2	0	198	61
Type-3	0	33	30
Type-4	0	18	17
Type-5	0	0	20
Type-6	0	2	6
Type-7	3	0	9
Type-8	13	4	10
Type-9	4	1	9
Type-10	1	0	0

Table S13: Genus-wise distribution of IRDs in dsBond types

	No. of IRDs for dsBond type									
**Genus**	**1**	**2**	**3**	**4**	**5**	**6**	**7**	**8**	**9**	**10**
**Capsicum**	86	58	17	10	0	0	2	9	2	1
**Nicotiana**	111	128	7	11	0	0	1	4	4	0
**Solanum**	51	33	38	12	0	2	0	8	4	0
**Non-Solanaceae**	1	41	1	2	20	6	9	5	5	0
